# Gene discovery, evolutionary affinity and molecular detection of *Oxyspirura petrowi*, an eye worm parasite of game birds

**DOI:** 10.1186/1471-2180-13-233

**Published:** 2013-10-21

**Authors:** Lixin Xiang, Fengguang Guo, Haili Zhang, Lloyd LaCoste, Dale Rollins, Andrea Bruno, Alan M Fedynich, Guan Zhu

**Affiliations:** 1 Department of Veterinary Pathobiology, College of Veterinary Medicine & Biomedical Sciences, Texas A&M University, College Station, Texas, USA; 2 College of Life Sciences, Zhejiang University, Hangzhou, Zhejiang, China; 3 Texas A&M AgriLife Research, San Angelo, Texas, USA; 4 Rolling Plains Quail Research Ranch, Rotan, Texas, USA; 5 Caesar Kleberg Wildlife Research Institute, Texas A&M University-Kingsville, Kingsville,, Texas, USA

## Abstract

**Background:**

*Oxyspirura petrowi* appears to be emerging as a nematode parasite that could negatively impact Northern Bobwhite quail individuals and populations within Texas and other regions of the United States. Despite this eye worm's potential importance in the conservation of wild quail, little is known about the general biology and genome composition of *O*. *petrowi*. To fill the knowledge gap, we performed a small scale random genome sequence survey, sequenced its 18S rRNA and the intergenic region between the 18S and 28S rRNA genes, studied its phylogenetic affinity, and developed a PCR protocol for the detection of this eye worm.

**Results:**

We have generated ~240 kb of genome sequence data derived from 348 clones by a random genome survey of an *O*. *petrowi* genomic library. The eye worm genome is AT-rich (i.e., 62.2% AT-content), and contains a high number of microsatellite sequences. The discovered genes encode a wide-range of proteins including hypothetical proteins, enzymes, nematode-specific proteins. Phylogenetic analysis based on 18S rRNA sequences indicate that the Spiruroidea is paraphyletic, in which *Oxyspirura* and its closely related species are sisters to the filarial nematodes. We have also developed a PCR protocol based on the ITS2 sequence that allows sensitive and specific detection of eye worm DNA in feces. Using this newly developed protocol, we have determined that ~28% to 33% of the fecal samples collected from Northern Bobwhites and Scaled Quail in Texas in the spring of 2013 are *O*. *petrowi* positive.

**Conclusions:**

The *O*. *petrowi* genome is rich in microsatellite sequences that may be used in future genotyping and molecular fingerprinting analysis. This eye worm is evolutionarily close to the filarial nematodes, implying that therapeutic strategies for filariasis such as *Loa loa* would be referential in developing treatments for the Thelazoidea parasites. Our qPCR-based survey has confirmed that *O*. *petrowi* infection is of potential concern to quail managers in Texas.

## Background

**Figure 1:**
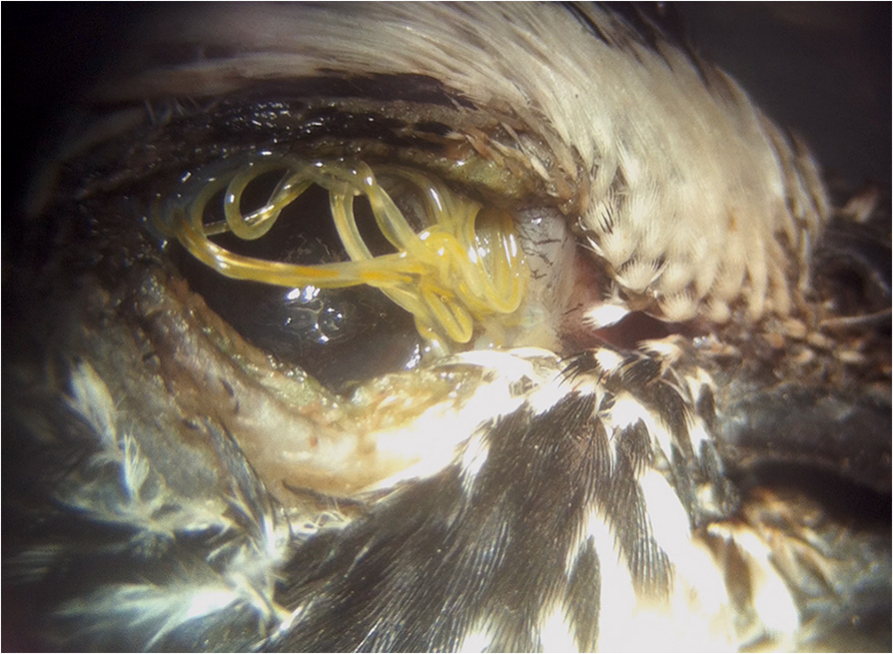
***Oxyspirura petrowi *** **adult worms in the eye of a Northern Bobwhite collected in Texas in February, 2013 demonstrating their potential to cause visual obstruction in addition to a pathological response resulting from infection.**

*Oxyspirura petrowi* is a spirurian nematode (Order Spirurida) that infects the eyes of quail and other birds [1]. In Texas, a 47–56% prevalence has been reported in Northern Bobwhites (*Colinus virginianus*) and Scaled Quail (*Callipepla squamata*) [2-4]. Similar infections caused by this genus of parasites have also been reported in other animals including poultry and zoo animals, where some of them were described as ocular oxyspiruriasis or oxyspirurosis [5-10]. Given that bobwhites are experiencing long-term declines throughout their range in North America, there is a recognition that populations are declining even where suitable habitat conditions exist (e.g., Rolling Plains ecoregion of Texas), thereby raising concerns that parasites such as *O*. *petrowi* may be a contributing factor (e.g., see a more detailed description at http://www.quailresearch.org). It is likely that infection may cause host eye damage and physically impair vision, making birds less competitive in feeding and more susceptible to predators (Figure 1).

Although the eye worm has been considered as a possible contributing factor for the decline of wild quail populations in the Rolling Plains, little is known of the parasite’s biology, particularly at the molecular and genomic levels (i.e., no molecular data were available in the GenBank databases prior to this study). Previous knowledge on the relationship of this parasite with other nematodes was solely acquired by morphology, which also needs to be validated at the molecular level. In fact, only a single nucleotide sequence is present in the database for the whole genus *Oxyspirura* (i.e., a 689-bp partial rRNA gene from *O*. *conjuctivalis* [GenBank:EF417873]). The lack of molecular data severely hampers our efforts in studying molecular epidemiology and transmission routes of *O*. *petrowi*, which may be useful for developing effective strategies to treat and control ocular oxyspiruriasis in wild quail.

To fill the knowledge gap, we have performed a small-scale genome sequence survey (GSS) that provides the first batch of genomic sequence data for this nematode. Additionally, we have cloned the 18S rRNA, internal transcribed spacer 1 (ITS1), 5.8S rRNA, ITS2 and partial 28S rRNA genes. The small random GSS effort rapidly generated ~240 kb of sequence information that provided not only a snapshot of the quail eye worm genome, but also a large amount of microsatellite sequences for future genotyping and population genetic analysis. Based on the newly obtained rRNA sequences, we determined the evolutionary affinity of *Oxyspirura* with other nematodes among spirurians with available 18S rRNA sequences. We also designed specific primers based on the ITS2 sequences, and performed real-time quantitative PCR (qPCR)-based molecular detection of *O*. *petrowi* from DNA extracted from fecal samples collected from Northern Bobwhite and Scaled Quail in Texas.

Understanding the biology of this parasitic nematode at molecular levels will enable us to effectively determine the prevalence by detecting parasite-specific DNA in feces, as well as to identify infected intermediate hosts that is otherwise difficult (if not impossible) based on morphology of larval stages. Molecular tools would enable further study of potential drug targets and target-based drug discovery to treat this important nematode.

## Methods

### Isolation of genomic DNA and genome sequence survey

Adult *O*. *petrowi* worms were isolated from the eyes of Northern Bobwhites collected in Texas as part of a 3-year integrated research project called Operation Idiopathic Decline, which was initiated to further our understanding of potentially pathogenic parasites occurring within the Rolling Plains Ecoregion of Texas and western Oklahoma. All animal experiments were performed in accordance with procedures approved by the Institutional Animal Care and Use Committee of Texas A&M University (protocol # 2011–193).

After microscopic examination for species validation, four worms were rinsed with PBS, placed in lysis buffer of the DNeasy Blood & Tissue Kit (Qiagen Inc., Valencia, CA), and grinded with a plastic microtube grinder. Genomic DNA (gDNA) was isolated from the ground worms according to manufacturer’s protocol for animal tissues. For the construction of a genomic library, gDNA was first subjected to whole genome amplification using a GenomePlex Complete Whole Genome Amplification (WGA) kit according the manufacturer’s standard protocol (Sigma-Aldrich Co., St. Louis, MO). Amplified gDNA products were fractionated in agarose gels and fractions containing fragments between 0.5 – 2 kb were collected and purified using a Gel Extraction Kit (Omega Bio-Tek, Norcross, GA). After an incubation at 72°C for 20 min in a regular PCR reaction buffer to add a single adenine overhang to the 3’-end, the products were ligated into pCR2.1-TOPO vector using a TOPO-TA cloning kit (Invitrogen, Life Technologies, Grand Island, NY).

After transformation, *Escherichia coli* OneShot TOP10F' chemically competent cells (Invitrogen) were plated onto LB plates spread with 40 μL of 40 mg/mL X-gal and 5 μL of 200 mM/mL IPTG, and incubated at 37°C overnight. Bacteria from a single white colony were collected into 10 μL Milli-Q water in a microtube, from which 2 μL of suspension was used directly as template in PCR reactions to determine the presence of inserts using a pair of M13 forward and M13 reverse primers. Colonies containing inserts with desired sizes were further incubated in LB broth containing 50 μg/mL kanamycin. Plasmids were prepared using a Plasmid DNA Mini kit (Omega Bio-Tek), and sequenced by the Sanger dye-terminator sequencing method at the Gene Technology Laboratory at Texas A&M University.

Vector and adaptor sequences were removed using a cross-match algorithm, and long inserts were assembled using the Phrap method implemented in the MacVector program (version 12.7.4) (http://www.macvector.com). All sequences were used as queries to search the non-redundant protein and nucleotide databases at the National Center for Biotechnology Information (NCBI) by the BLASTN, BLASTX and TBLASTX algorithms using the KoriBlast program (version 3.4) (http://www.korilog.com). Additional annotations were performed using the Blast2GO program (http://www.blast2go.com/b2ghome), which included InterProScan for identifying protein domains and gene ontology (GO) analysis. GO_slim was performed at the CateGOrizer server (http://www.animalgenome.org/cgi-bin/util/gotreei) [11]. Contigs were also mapped onto the metabolic pathways at the Kyoto Encyclopedia of Genes and Genomes (KEGG) using the KEGG Automatic Annotation Server (KAAS) (http://www.genome.jp/tools/kaas/) [12]. Candidate tRNA sequences were examined at the tRNAscan-SE server (http://lowelab.ucsc.edu/tRNAscan-SE/) [13]. Microsatellite sequences (also known as simple sequence repeats, SSRs) were identified using the Phobos (version 3.3.12) program (http://www.ruhr-uni-bochum.de/spezzoo/cm/cm_phobos.htm), in which only perfect matches with a minimal length of 8 nt and a minimal score at 8 were reported.

### Molecular cloning of parasite ribosomal RNA (rRNA) genes

The 18S rRNA gene and downstream ITS1, 5.8S rRNA and ITS2 regions from *O*. *petrowi* were cloned by PCR using two pairs of primers: 1) nema18S_F01 (5’-CCA TGC AWG TCT AWG TTC AAA-3’) and nema18S_R01 (5’-GGA AAC CTT GTT ACG ACT TTT G-3’) for the nearly whole 18S region; and 2) nema18S_F1400 (5’-GTC TGT GAT GCC CTT AGA TG-3’) and nema28S_R68 (5’-TTA GTT TCT TTT CCT CCG CTT A-3’) for the region between the 18S and 28S rRNA genes. PCR was performed using a JumpStart REDTaq ReadyMix PCR Reaction kit containing hot-start high-fidelity DNA polymerase (Sigma-Aldrich). After treating with regular Taq DNA polymerase at 72°C for 10 min, PCR amplicons were similarly cloned into the pCR2.1-TOPO vector as described above. At least 10 independent clones from each reaction were sequenced, and all reads were assembled by Phrap as described above. Regions representing 18S, ITS1, 5.8S, ITS2 and partial 28S sequences were determined by Rfam (http://rfam.janelia.org) [14].

### Phylogenetic reconstructions

The assembled *O*. *petrowi* 18S rRNA sequence was used as a query to search and identity nematode orthologs from the NCBI nucleotide databases. Up to 1,000 gene sequences were initially retrieved, subjected to multiple sequence alignments using the MUSCLE program (version 3.8.31) (http://drive5.com/muscle/) [15,16], followed by simple phylogenetic reconstructions by the neighbor-joining (NJ) method using the Tamura-Nei nucleotide substitution model. Based on the alignment and NJ trees, short or identical sequences were individually removed, and the same procedure was repeated until a balanced dataset containing 111 sequences representing all major nematode taxonomic groups were identified. The dataset was subjected to phylogenetic reconstructions by Bayesian inference (BI) using MrBayes (version 3.2) (http://mrbayes.sourceforge.net) and the maximum likelihood (ML) method using TreeFinder (version 2008) (http://www.treefinder.de) [17,18]. This approach determined the phylogenetic relationships among major taxonomic groups, in which *O*. *petrowi* was placed within the spirurians, but the relationship among spirurians was not well resolved. Therefore, we resampled the sequences to include only taxa within Spirurida and Ascaridida as these two groups displayed a sister relationship by this study and previous analyses [19,20]. This also allowed us to include more taxa within these two groups. The second dataset contained 112 taxa with 1,544 nucleotide positions and was subjected to phylogenetic reconstructions using BI and ML methods. To further resolve the *O*. *petrowi* position, we also compiled a third dataset containing only taxa with close relationship with *O*. *petrowi*. This small dataset included only 35 taxa with 1,599 nucleotide positions, and was also subjected to BI and ML analyses.

In all datasets, gaps were removed and only positions that could be unambiguously aligned were used in subsequent phylogenetic analyses. In the BI analysis, 1.5 million generations of searches for the first and second datasets (or 1.0 million generations for the smaller third dataset) were performed with 4 independent chains running. Searches reached convergence as determined by the average standard deviation (SD) of split frequencies reaching < 0.01, and potential scale reduction factor (PSRF) values for various approaching 1.0 [21]. Bootstrapping ML analyses were derived from 200 replicated sequences. In both BI and ML methods, the general time reversal (GTR) nucleotide substitution model was used with the consideration of fraction of invariance and 4-rate of discrete gamma (i.e., **GTR** + ***F***_**inv**_ + ***Γ***_**4**_). Majority rule consensus trees were visualized using FigTree (version 1.4), followed by tree annotations using Adobe Illustrator CS4.

### Molecular detection of *O*. *petrowi*

Sequence comparison of the rRNA regions between *O*. *petrowi* and other nematodes indicated that 18S rRNA sequences were less suitable for designing species-specific primers, as they were highly conserved among nematodes. We hence designed primers based on the ITS2 region sequences for specific molecular detection for *O*. *petrowi*: QEW_2417F (5’-GGA TTT GCA AGA ATT GTT TCC-3’) and QEW_2578R (5’-AAC GTT ATT GTT GCC ATA TGC-3’) with a predicted product size of 162 bp. To ensure desired specificity of detection, we used DNA samples isolated from *Aulonocephalus pennula*, a cecal worm collected from bobwhite quail in northern Texas as negative control.

We applied real-time quantitative PCR (qPCR) to detect eye worm DNA from fecal samples from Northern Bobwhite and Scaled Quail in Texas. Feces from individual or pooled birds were collected at Rolling Plains Quail Research Ranch (RPQRR) in Fisher County, Texas in the Spring, 2013 via the seasonal trap-and-release program in a separate conservation research project. Feces were mixed, weighed and placed in lysis buffer included in the QIAamp DNA Stool Mini Kit (Qiagen). After one freeze/thaw cycle in liquid nitrogen, samples were homogenized with glass beads in a Mini-Beadbeater-16 (BioSpec Products, Inc., Bartlesville, OK) at full-speed for 4 min, followed by DNA isolation according to the manufacturer’s protocol for the stool DNA isolation kit. A SYBR-green-based qPCR detection was performed in 20 μL reactions containing iQ SYBR Green Supermix reagent (Bio-Rad, Hercules, CA), the QEW_2417F/QEW_2578R primer pair (each at 100 nM) and 2 μL stool DNA in a Bio-Rad iCycle iQ Real-Time PCR Detection System. The reactions started with sample denaturation at 95°C for 5 min, followed by 45 thermal cycles at 95°C for 30 sec, 56°C for 30 sec, and 72°C for 30 sec. Specificity of detection was confirmed by melting curve analysis and agarose gel electrophoresis of selected PCR products. Selected individual or pooled PCR products were also sequenced to confirm their identities.

### Nucleotide sequence accession numbers

Nucleotide sequences generated in this study were deposited into the GenBank database with accession numbers [GenBank:KF110799] and [GenBank:KF110800] for rRNA sequences containing type 1 and type 2 ITS1, and [GenBank:KG007611] to [GenBank:KG007945] for GSS sequences.

## Results and discussion

### Characterization of the *O*. *petrowi* genome

In this small genome sequence survey, we have obtained valid sequences for 354 clones. Among them, six sequences were determined to be bacterial contaminants, representing 1.6% of the sequenced clones. There were no contaminants from birds and other organisms, suggesting that the prepared *O*. *petrowi* genomic libraries were of high quality. The limited bacterial sequences (top hits were mainly *Ralstonia* and *Caulobacter vibrioides*) were likely derived from commensal bacteria in *O*. *petrowi*.

The remaining 348 valid *O*. *petrowi* sequences resulted in 237,239 bp of genomic sequences, which ranged from 81 to 1,220 bp (median length = 706 bp) for individual contigs. The scale of the survey was relatively small, but it was sufficient to provide a first snapshot of the genome features for this nematode. The *O*. *petrowi* genome was generally AT-rich with GC contents ranging from 18% to 64% for individual contigs (overall mean of GC content = 37.8%, vs. 56% to 70% for the six bacterial contaminants). Various BLAST searches yielded no hits for 137 contigs (~39%), suggesting that these sequences might be unique to *Oxyspirura* or closely related species. Among the 211 contigs with hits from the databases, 193 (91.5%) had top hits on nematode sequences, particularly on those from *Loa*, *Brugia* and *Wuchereria*, but only very few on *Ascaris* or *Caenorhabditis*, which was congruent with the evolutionary relationship of *Oxyspirura* with filarioidea, Ascaridida and Rhabditida (see Additional file 1: Table S1 for a complete list of all contigs with annotations and corresponding BLAST top hits).

**Figure 2:**
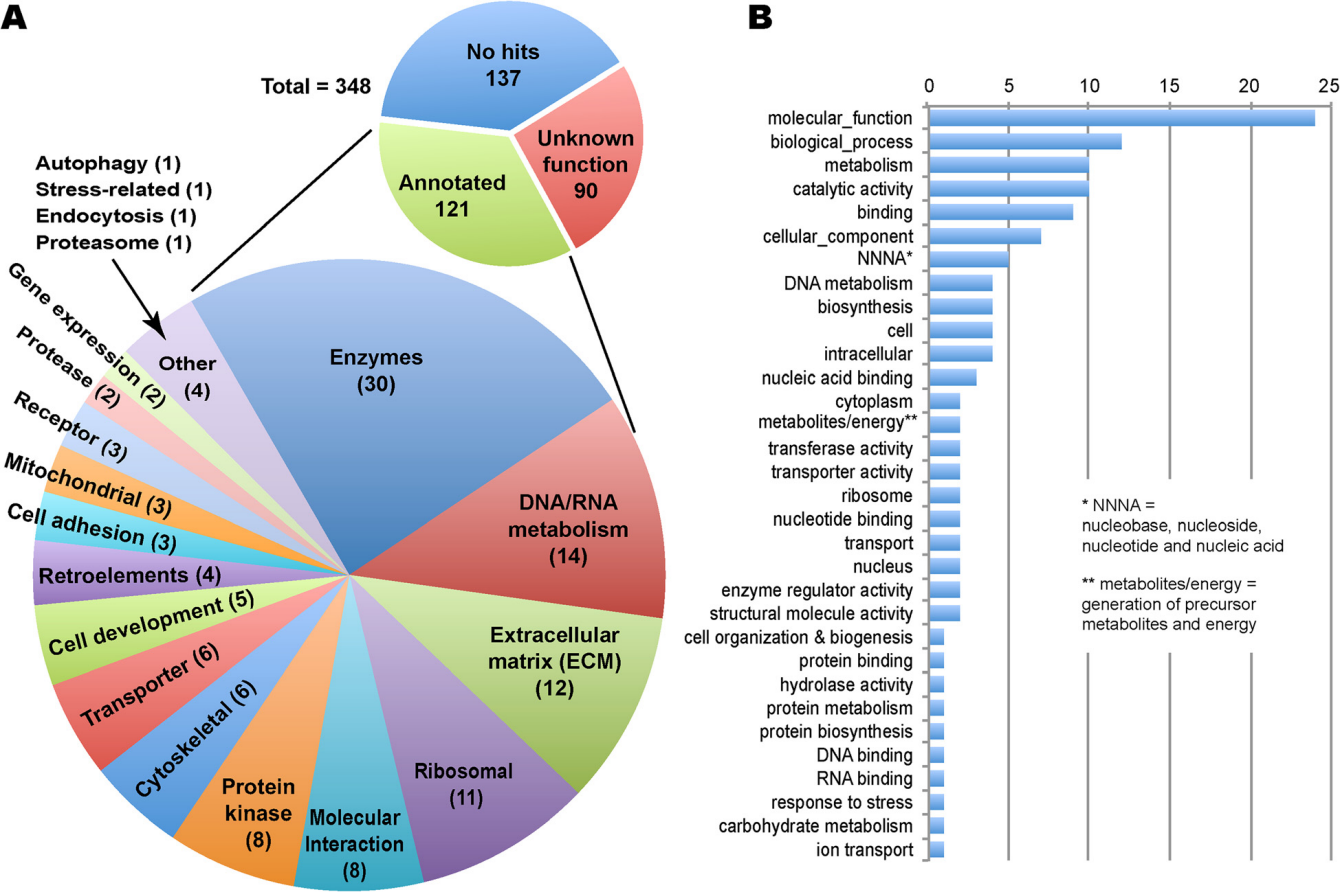
**Classification of *Oxyspirura petrowi *genes discovered by the random genome sequence survey by major functional groups (A) or gene ontology (GO) terms (B).** A list of gene contigs with annotations is provided in Additional file 1: Table S1.

By combining BLAST with InterProScan searches, more than half of the contigs with hits were able to be assigned into major functional categories (i.e., 121 out of 211 contigs) (Figure 2). The functionally undefined 90 contigs were mainly hypothetical proteins with some containing low complexity sequences. Among the 121 annotatable contigs, the largest group was enzymes (total 40) that were separated into general enzymes involved in various metabolic pathways (n = 30) and those involved in protein metabolism such as protein kinases (n = 8) and proteases (n = 2). Examples of enzymes included glycogen synthase (contig QEW_195), glycosyl-transferase (QEW_224), histone acetyltranferase (QEW_156), and succinate dehydrogenase (QEW_315); while those for protein metabolism included a Ulp1 protease family member (QEW_129), as well as protein kinases in the AGC/NDR (QEW_74), CAM/CAMKL/NUAK (QEW_372) and CK1/WORM6 (QEW_249) families. Fourteen contigs encoded proteins involved in DNA/RNA metabolism, e.g., splicing factor 1 (QEW_306), DNA replication licensing factor mcm-6 (QEW_340), and protein containing double-stranded RNA binding motif (QEW_379). There were 12 contigs containing genes encoding extracellular membrane (ECM) proteins, in which 9 contigs were associated with the nematode-specific cuticle formation, such as cuticle collagen precursors (QEW_58, QEW_59 and QEW_135), cuticulin-1 (QEW_104), and nematode cuticle collage domain containing proteins (e.g., QEW_80 and QEW_386). Other groups include those involved in ribosomal biogenesis (n = 11), molecular interactions (n = 8), ion transporters (n = 6), cytoskeletal proteins (n = 6), membrane proteins associated with cell adhesion (n = 3), and those involved in gene expression (n = 2).

Interestingly, there were three contigs encoding nematode-specific major sperm protein (MSP), which was grouped together with a fic protein under the “cell development” category in Figure 2. There were also three contigs derived from the mitochondrial genomes, including gene fragments encoding cytochrome b, cytochrome c oxidase subunit IV (COX-IV) and NAHD dehydrogenase subunit 5. Finally, 4 contigs were found to contain retroelements, such as tigger transposable element-derived protein 1-like proteins (QEW_112 and QEW_119) and retrotransposon protein (QEW_172).

### Microsatellite sequences identified by the genome survey

One major purpose of the study was to identify microsatellite sequences (also known as simple sequence repeats, SSRs) for future genotyping and population genetics analyses on this quail eye worm. In fact, *O*. *petrowi* appears to be rich in microsatellites, in which a total of 335 units of perfect SSRs were identified with a minimal length of 8 nt (Table 1). These included mononucleotides (228 units), dinucleotides (30), trinucleotides (56), tetranucleotides (11) and 10 repeats with 5–8 nucleotides. At least 98 contigs contained two or more SSRs, and 31 contigs contained 3–6 SSRs (Table 1). Examples included QEW_123 with 5 for mono-, tri- or tetra-nucleotide SSRs; QEW_126 with 5 mono-, tetra- or octa-nucleotide SSRs, and QEW_203 with 6 di-, tri- or penta-nucleotide SSRs (see Additional file 2: Table S2 for a complete list of detected microsatellite sequences). We also looked at the distribution of microsatellites with repeat units of ≥2 nt, which revealed ~2 or ~1.5 times more microsatellite sequences are present in contigs with no hits in BLAST/InterProScan searches (19.0%) or with hits but unknown function (14.4%) than in the annotatable contigs (9.9%) (Table 2). In summary, the eye worm genome contains a rich number of microsatellite sequences with the potential to be further validated as potential genetic markers.

**Table 1:** **Statistics on the lengths of repeat units and numbers of microsatellite sequences per contig in *Oxyspirura petrowi *** **identified by the genome sequence survey**

**Length of repeat unit**	**Counts**	**No. microsatellites per contig**	**Counts**
1	228	1	86
2	30	2	67
3	56	3	17
4	11	4	7
5	2	5	6
6	6	6	1
8	2	≥7	0
**Total microsatellites**	**335**	**Average no. per contig**	**1.82**

**Table 2:** Number of microsatellites (SSR) with unit length ≥2 by functional groups*

**Group**	**No. contigs**	**SSR (unit > =2)**	**Percentage**
Annotatable	121	12	9.9%
Function unknown	90	13	14.4%
No hits	137	26	19.0%
Total	348	51	14.7%

* See Additional file 2: Table S2 for a complete list of microsatellite sequences.

### Phylogenetic position of *O*. *petrowi* based on 18S rRNA genes

Our first phylogenetic analyses based on a large 18S rRNA dataset with BI and ML methods produced trees that agreed with those produced by others. While *O*. *petrowi* was clustered within the Spirurida clade, it was close to a branch consisting of *Tetrameres fissipina* and an unknown Onchoceridae species. This was likely a result caused by a long branch attraction (LBA) artifact based on the unusual long branch formed by *T*. *fissipina* and the Onchoceridae species, as well as by the obvious high numbers of nucleotide substitutions in these two sequences (data not shown). We also observed potential sequencing mistakes for the long 18S rRNA sequence of *Thelazia lacrymalis* (DQ503458). Therefore, we removed these three sequences from subsequent analyses. Because Spirurida was consistently placed as a sister group with Ascaridida, as observed in this study and by others [19,20], our second dataset contained only sequences from these two groups, which allowed more reliable alignment with more nucleotide positions to better resolve the position of *O*. *petrowi* within Spirurida using Ascaridida as outgroup. *Gnathastoma* sequences were also excluded from the second dataset, as they have been shown to be seperate from the rest of the spirurids [19,20].

**Figure 3:**
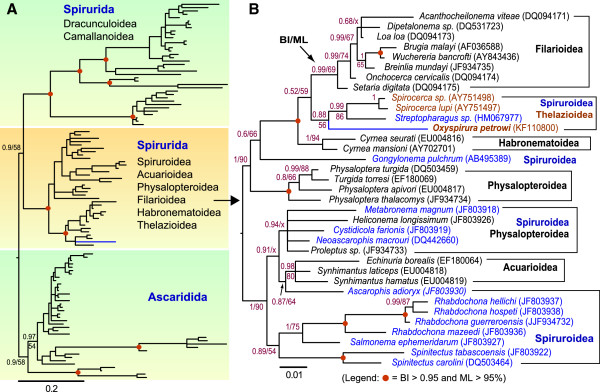
**Phylogenetic relationship of *Oxyspirura petrowi *within the Spirurida nematodes as determined by Bayesian inference (BI) and maximum likelihood (ML) methods based on 18S rRNA sequences from Spirurida and Ascaridida (112 taxa with 1,544 positions) (A) and from species more closely related to Thelazioidea (35 taxa with 1,599 positions) (B).** In both approaches, the general time reversal (GTR) nucleotide substitution model was used with the consideration of fraction of invariance and 4-rate of discrete gamma (i.e., **GTR** + ***F***_**inv**_ + ***Γ***_**4**_). Numbers at the nodes indicate posterior probability (BI) and bootstrap proportion (ML) supporting values. Nodes highlighted by dots were supported by >95% in both BI and ML bootstrapping analyses. Letter “x” indicates nodes supported by <50% in either BI or ML analysis.

Both BI and ML trees inferred from the second dataset distinctly separated Ascaridida from Spirurida (Figure 3A). Within the Spirurida clade, Dracunculoidea and Camallanoidea formed two major sister branches, whereas the third branch comprised of the remaining families including Spiruroidea, Acuarioidea, Physalopteroidea, Filarioidea, Habronematoidea and Thelazioidea. Further phylogenetic analysis based only on sequences from the third branch produced similar tree topology, but with slightly better resolution and statistical support (Figure 3B). Acuarioidea, Physalopteroidea, Filarioidea and Habronematoidea were monophyletic, whereas Spiruroidea was paraphyletic, intermixed with other families. Among them, *O*. *petrowi* was clustered with *Streptopharagus* and *Spirocerca*, which in turn formed a sister branch to the Filarioidea, albeit with low posterior probability and bootstrap proportion support (Figure 3B). At the moment, more sophisticated phylogenetic analyses were unachievable due to the lack of more sequences from closely related species, and the lack of sufficient sequence data such as the mitochondrial genomes and proteins within Spirurida, particularly among Thelazioidea. Nonetheless, our study revealed that Thelazioidea, including quail eye worm, was closely related to filarial nematodes, which implies that therapeutic strategies for filariasis such as those for *L*. *loa* might be referential in developing treatments for the Thelazoidea eye worms.

### Feature of internal transcribed regions and molecular detection of *O*. *petrowi*

**Figure 4:**
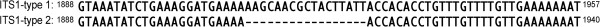
**Comparison of the *Oxyspirura petrowi *type 1 and type 2 ITS1 sequences at the insertion/deletion site.** Their sequences are available at GenBank database with accession numbers [GenBank:KF110799] and [GenBank:KF110800].

In addition to the nearly complete 18S rRNA gene, we have also determined the complete sequences of the ITS1, 5.8S rRNA and ITS2 regions. Two types of ITS1 sequences have been observed (GenBank accession numbers: KF110799 for type 1 and KF110800 for type 2 ITS1), in which the type 1 sequence contained a 17-nucleotide insertion at positions 1911 – 1927 (Figure 4). Additionally, there were two nucleotide differences between the two types of ITS1 sequences at positions 2112 and 2188 (in reference to KF110799). The insertion/deletion was further confirmed by PCR using primers flanking the region and subsequent sequencing of PCR amplicons.

**Figure 5:**
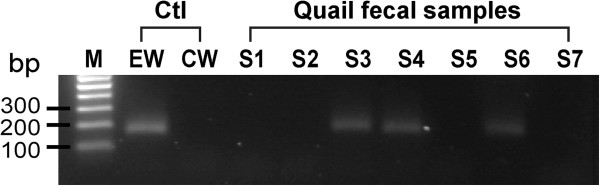
**Agarose gel (1.5%) electrophoresis illustrating PCR-based detection of *Oxyspirura petrowi *DNA from quail fecal samples.** Lane M: 100-bp molecular marker; Lanes EW and CW: regular PCR using DNA isolated from adult eye worm (EW, *O*. *petrowi*) and cecal worm (CW, *A*. *pennula*) isolated from wild quail as positive and negative controls (Ctl); Lanes S1 – S7: results of real-time qPCR detection from selected positive and negative stool DNA samples.

While the 5.8S rRNA gene shared significant sequence homology to those of other nematodes available the NCBI databases (data not shown), both the ITS1 and ITS2 regions displayed high sequence diversity (i.e., no significant hits in BLASTN searches of the NCBI nucleotide databases). Because of the insertion/deletion at the ITS1 region, we initially designed two pairs of primers based on the ITS2 sequence for the potential of using nested PCR-based molecular detection of *O*. *petrowi*: an external primer pair QEW_2373F (5’-AAG AAT GTA ATG TTG TGG AGC-3’) and QEW_2681R (5’-GTA ATC ACA TTT GAG TTG AGG-3’), and an internal primer pair QEW_2417F and QEW_2578R (as described in the Methods section) that would give 309 bp and 162 bp products, respectively. However, after our pilot experiments indicated that nested PCR was unnecessary, we used regular qPCR reactions with the QEW_2417F and QEW_2578R primers in subsequent detection of *O*. *petrowi* DNA from wild bobwhite fecal specimens collected in Texas in February – March, 2013. The specificity of the QEW_2417F and QEW_2578R primer pair for *O*. *petrowi* was also confirmed by its inability to produce products from DNA isolated from the cecal worm *A*. *pennula* that is commonly present in wild quail (Figure 5).

On the other hand, due to the lack of molecular sequences at the ITS regions from very closely related species, we could not firmly conclude that the relatively short primers were absolutely specific to *O*. *petrowi*. In fact, only six ITS1 sequences were present in the GenBank database for the superfamily Thelazioidea, including five from *Thelazia* species and one from *O*. *conjuctivalis* (accession: EF417873). However, these ITS1 sequences were highly divergent from each other and from those of *O*. *petrowi* (i.e., 47.5% - 48.5% identities between the *O*. *conjuctivalis* and *O*. *petrowi* and 26.1% - 53.2% between the five *Thelazia* species within the ITS1 sequences), which indicated that their ITS2 regions were likely divergent enough for speciation. To further ensure the quality of detection, selected individual or pooled PCR products were also sequenced to validate their identities. When the qPCR detection system was used, melting curve analysis was performed to confirm that PCR amplicons showed the same curves as those of positive controls.

Among the 54 fecal DNA specimens, we have detected 21 (38.9%) positive samples. However, because the specimens were collected from both individual and pooled quail samples derived from 88 birds, direct calculation of positive rate (i.e., 21/54 = 38.9%) was inappropriate as pooled positive specimens might contain both positive and negative samples. Therefore, we employed two additional approaches to estimate the prevalence. The first approach was to only calculate positive rate from the 39 samples collected from individual birds (non-pooled), in which 13 samples were positive, giving a 33.3% positive rate. The second approach employed software written by Dr. Brad Biggerstaff as an Excel Add-In (PooledInfRate, version 3.0) (http://www.cdc.gov/westnile/resourcepages/mosqSurvSoft.html), which was originally developed to determine positive rates of viral infections in pooled mosquito samples using a maximum likelihood estimation (MLE) algorithm [22]. By applying a bias-corrected MLE estimation, we obtained an infection rate of 27.7% with lower and upper limits at 18.6% and 38.7%, respectively (95% confidence interval). Collectively, we conclude that ~30% (or between 28% - 33%) of the sampled wild quail were infected by the eye worm. The actual rate might be even higher, as the fecal samples were only collected once, rather than continuously for several days, and the sensitivity of PCR detection might not be maximal due to the inhibitory substances commonly present in fecal samples as discussed below.

The detection of *O*. *petrowi* DNA in feces allows rapid and sensitive detection of the presence of eye worms without the need to examine individual birds. However, one needs to be aware of the presence of inhibitory substances in fecal samples and the difficulties in releasing DNA from eggs or encysted larvae. The presence of inhibitory substances could be minimized (if not completely eliminated) using the tablets included in the DNA isolation kits specifically designed for stool samples such as the QIAamp DNA Stool Mini Kit (Qiagen). Freeze/thaw cycles combined with homogenization with glass beads were necessary to break the eggs or encysted larvae to ensure the release of DNA. Furthermore, nested PCR might also be used by the addition of a primary amplification using the external primers QEW_2373F and QEW_2681R to not only improve the sensitivity of PCR detection, but to also further eliminate the presence of inhibitory substances in a second amplification procedure.

The life cycle of *O*. *petrowi* is not well understood, its exact intermediate host(s) as well as its migration details in quail. The presence of *O*. *petrowi* DNA in quail feces indicates that eggs laid by female adult worms in the eyes could be passed into the digestive system and released with the excrement via the naso-lacrimal ducts to the gut route, as previously observed in other eye worms such as *O*. *mansoni*[23].

This PCR detection protocol can also be easily adapted to identify intermediate host(s) and perform surveillance, which is important in developing effective strategies to control the transmission of eye worms in the field. Our phylogenetic analysis based on 18S rRNA sequences indicated that *O*. *petrowi* clustered closely with *Streptopharagus* and *Spirocerca* (Figure 3). It is known that birds are a paratenic host for *Spirocerca lupi* infection in their life cycle between dogs and dung beetles [24]. Dung beetles are also the intermediate host for *Streptopharagus*[25]. These observations suggest that dung beetles might be worth examining as one of the potential intermediate hosts. Indeed, the detection of *O*. *petrowi* DNA in various insects including dung beetles is currently ongoing as part of a separate project in determining the intermediate host(s) and transmission route(s), and the data will be reported upon the completion of the survey.

## Conclusions

We have performed a small-scale genome sequence survey (GSS), which not only rapidly generated a large number of molecular sequence data for the first time for *O*. *petrowi*, but also provided a snapshot of the genome for the eye worm in quail. The survey also identified a large number of microsatellite sequences that may be employed in further genotyping and population genetics studies. Our phylogenetic reconstructions based on 18S rRNA sequences indicated that Spiruroidea was paraphyletic, while *O*. *petrowi*, *Streptopharagus* and *Spirocerca* formed a sister clade to the filarial nematodes. The obtained ITS sequence data also permitted us to design specific primers for molecular detection of *O*. *petrowi* in fecal samples, which may also be adapted to detect this nematode in insect intermediate hosts for surveillance and developing strategies to control the transmission of eye worms from intermediate hosts to quail. We also determined that ~28% - 33% of the birds were *O*. *petrowi* positive, suggesting that eye worm was a significant parasite in at least some quail ranches in Texas.

## Competing interests

The authors declare that they have no competing interests.

## Authors’ contributions

LX participated in experimental design and performed the majority of experiments on the genome survey including constructing genomic library, cloning and sequencing, the cloning and sequencing of rRNA gene and downstream region sequences, and the isolation stool DNA and PCR/qPCR detection; FG and HZ participated in sample preparation; LL participated in collection of fecal samples from wild quail; AB participated in collection of adult eye worms; DR participated in fecal sample collection, writing the manuscript, and securing funding for the study; AMF participated in collection and speciation of eye worm and writing manuscript; GZ conceived the study, participated in its design, molecular and phylogenetic analysis, and writing the manuscript. All authors read and approved the final manuscript.

## Supplementary Material

Additional file 1: Table S1 List of contigs with annotations and information on top blast hits. Click here for file

Additional file 2: Table S2 *Oxyspirura petrowi* microsatellite sequences identified by the GSS (all perfect matches) using Phobos. Click here for file

## References

[B1] Pence DB The genus *Oxyspirura* (nematoda: thelaziidae) from birds in Louisiana Proc Helminth Soc Washington 1972 13 1 23 28

[B2] Landgrebe JN Vasquez B Bradley RG Fedynich AM Lerich SP Kinsella JM Helminth community of scaled quail (*Callipepla squamata*) from western Texas J Parasitol 2007 13 1 204 208 17436967 10.1645/GE-3578RN.1

[B3] Jackson AS A handbook for bobwhite quail management in west Texas rolling plains 1969 Texas Parks: Wildlife Department

[B4] Villarreal SM Helminth infections across the annual breeding cycle of northern bobwhites from Fisher County, Texas 2012 Kingsville, TX: Texas A&M University-Kingsville

[B5] Addison EM Prestwood AK *Oxyspirura turcottei* n.sp. (Nematoda: Thelaziidae) from the eastern wild turkey (*Meleagris gallopavo* silvestris) Can J Zool 1978 13 5 1218 1221 667760 10.1139/z78-170

[B6] Ali SM On some new species of the genus *Oxyspirura* from birds in Hyderabad, Andhra Pradesh, India J Helminthol 1960 13 221 242 13682407 10.1017/s0022149x00021167

[B7] Ivanova E Spiridonov S Bain O Ocular oxyspirurosis of primates in zoos: intermediate host, worm morphology, and probable origin of the infection in the Moscow zoo Parasite 2007 13 4 287 298 18225417 10.1051/parasite/2007144287

[B8] Jairapuri DS Siddiqi AH A review of the genus *Oxyspirura* Drasche in Stossich, 1897 (Nematoda: Thelaziidae) with descriptions of fourteen new species J Helminthol 1967 13 4 337 363 6057047 10.1017/s0022149x00021891

[B9] Schwabe CW Studies on *Oxyspirura mansoni*, the tropical eyeworm of poultry. III. Preliminary observations on eyeworm pathogenicity Am J Vet Res 1950 13 40 286 290 15425755

[B10] Vellayan S Jeffery J Oothuman P Zahedi M Krishnasamy M Paramaswaran S Rohela M Abdul-Aziz NM Oxyspiruriasis in zoo birds Trop Biomed 2012 13 2 304 307 22735854

[B11] Hu ZL Bao J Reecy JM CateGOrizer: a Web-based program to batch analyze gene ontology classification categories Onl J Bioinform 2008 13 2 108 112

[B12] Moriya Y Itoh M Okuda S Yoshizawa AC Kanehisa M KAAS: an automatic genome annotation and pathway reconstruction server Nucleic Acids Res 2007 13 182 185 10.1093/nar/gkm321 PMC1933193 17526522

[B13] Schattner P Brooks AN Lowe TM The tRNAscan-SE, snoscan and snoGPS web servers for the detection of tRNAs and snoRNAs Nucleic Acids Res 2005 13 686 689 10.1093/nar/gki366 PMC1160127 15980563

[B14] Gardner PP Daub J Tate J Moore BL Osuch IH Griffiths-Jones S Finn RD Nawrocki EP Kolbe DL Eddy SR Rfam: wikipedia, clans and the "decimal" release Nucleic Acids Res 2011 13 141 145 10.1093/nar/gkq1129 PMC3013711 21062808

[B15] Edgar RC MUSCLE: a multiple sequence alignment method with reduced time and space complexity BMC Bioinforma 2004 13 113 10.1186/1471-2105-5-113 PMC517706 15318951

[B16] Edgar RC MUSCLE: multiple sequence alignment with high accuracy and high throughput Nucleic Acids Res 2004 13 5 1792 1797 15034147 10.1093/nar/gkh340 PMC390337

[B17] Jobb G von Haeseler A Strimmer K TREEFINDER: a powerful graphical analysis environment for molecular phylogenetics BMC Evol Biol 2004 13 18 15222900 10.1186/1471-2148-4-18 PMC459214

[B18] Ronquist F Teslenko M van der Mark P Ayres DL Darling A Hohna S Larget B Liu L Suchard MA Huelsenbeck JP MrBayes 3.2: efficient Bayesian phylogenetic inference and model choice across a large model space Syst Biol 2012 13 3 539 542 22357727 10.1093/sysbio/sys029 PMC3329765

[B19] Smythe AB Sanderson MJ Nadler SA Nematode small subunit phylogeny correlates with alignment parameters Syst Biol 2006 13 6 972 992 17345678 10.1080/10635150601089001

[B20] Meldal BH Debenham NJ De Ley P De Ley IT Vanfleteren JR Vierstraete AR Bert W Borgonie G Moens T Tyler PA An improved molecular phylogeny of the Nematoda with special emphasis on marine taxa Mol Phylogenet Evol 2007 13 3 622 636 17084644 10.1016/j.ympev.2006.08.025

[B21] Gelman A Rubin DB Inference from iterative simulation using multiple sequences Stat Sci 1992 13 4 457 472

[B22] Hepworth G Confidence intervals for proportions estimated by group testing with groups of unequal size J Agr Biol Envir St 2005 13 4 478 497

[B23] Schwabe CW Studies on *Oxyspirura mansoni*, the tropical eyeworm of poultry, II. Life history Pacific Sci 1951 13 1 18 35

[B24] Oryan A Sadjjadi SM Mehrabani D Kargar M Spirocercosis and its complications in stray dogs in Shiraz, southern Iran Vet Med 2008 13 11 617 624

[B25] Boze BGV Hernandez AD Huffman MA Moore J Parasites and dung beetles as ecosystem engineers in a forest ecosystem J Insect Behav 2012 13 4 352 361

